# Preparation of single- and double-oligonucleotide antibody conjugates and their application for protein analytics

**DOI:** 10.1038/s41598-020-58238-6

**Published:** 2020-01-29

**Authors:** Julius Wiener, Daniel Kokotek, Simon Rosowski, Heiko Lickert, Matthias Meier

**Affiliations:** 1 Microfluidic and Biological Engineering, Helmholtz Pioneer Campus, Helmholtz Zentrum München, Ingolstaedter Landstr. 1, 85764 Neuherberg, Germany; 2 Microfluidic and Biological Engineering, IMTEK, University of Freiburg, Georges-Koehler-Allee 103, 79110 Freiburg, Germany; 3 Institute of Diabetes and Regeneration Research, Helmholtz Zentrum München, D-85764 Neuherberg, Germany; 4 German Center for Diabetes Research (DZD), D-85764 Neuherberg, Germany; 5 Institute of Stem Cell Research, Helmholtz Zentrum München, D-85764 Neuherberg, Germany; 6 Technical University of Munich, School of Medicine, Munich, Germany

**Keywords:** Immunochemistry, Chemical modification, Biochemistry, Chemical biology, Analytical biochemistry, Biochemical assays, Antibody isolation and purification, Immunohistochemistry

## Abstract

Oligonucleotide-conjugated antibodies have gained importance for their use in protein diagnostics. The possibility to transfer the readout signal from the protein to the DNA level with an oligonucleotide-conjugated antibody increased the sensitivity of protein assays by orders of magnitude and enabled new multiplexing strategies. A bottleneck in the generation of larger oligonucleotide-conjugated antibody panels is the low conjugation yield between antibodies and oligonucleotides, as well as the lack of product purification methods. In this study, we combined a non-site-directed antibody conjugation technique using copper-free click chemistry with ion-exchange chromatography to obtain purified single and double oligonucleotide-conjugated antibodies. We optimized the click conjugation reaction of antibodies with oligonucleotides by evaluating crosslinker, reaction temperature, duration, oligonucleotide length, and secondary structure. As a result, we were able to achieve conjugation yields of 30% at a starting quantity as low as tens of nanograms of antibody, which makes the approach applicable for a wide variety of protein analytical assays. In contrast to previous non-site-directed conjugation methods, we also optimized the conjugation reaction for antibody specificity, confirmed by testing with knockout cell lines. The advantages of using single or double oligonucleotide-conjugated antibodies in regards to signal noise reduction are shown within immunofluorescence, proximity ligation assays, and single cell CITE-seq experiments.

## Introduction

Oligonucleotide-conjugated antibodies are commonly used in therapeutic cell targeting and protein diagnostics. One emerging therapeutic application involves exploiting the protein binding specificity of antibodies to deliver anti-sense oligonucleotides for the silencing of cell type-specific genes^1^. In diagnostics, oligonucleotide-conjugated antibodies are used to translate the detection signal from the protein to the DNA level^2^. This allows for the use of highly sensitive DNA amplification methods to amplify the readout signal. Prominent examples in this respect are immuno-PCR^3^ or hybridization chain reaction methods^4–6^ with oligonucleotide-conjugated antibodies, which has been proven to increase sensitivity by multiple magnitudes compared to standard ELISA technologies. The spectrum of applications for oligonucleotide-conjugated antibodies is expanding beyond protein interactions^7^ into enzyme activity^8^. Further, the transfer of the readout signal from protein to the DNA allows for the implementation of high multiplexing strategies for protein analytical assays^9–11^, since the target specificities of the antibodies can be encoded within the oligonucleotide sequences. Combined with next generation sequencing (NGS) technologies or sequential fluorescence hybridization methods, tens of proteins could be quantitated in parallel with e.g. CITEseq^12^. or SABER^13^.

One bottleneck in the development of antibody-oligonucleotide assay technologies consists of the conjugation reaction between the two biomolecules. Several protocols are available offering solutions for the conjugation of antibodies with oligonucleotides^13–17^. The most specific option for the preparation of antibody-oligonucleotide conjugates involves site-directed conjugation methods, where the oligonucleotide is conjugated to either n-linked glycans or internally-expressed protein tags^18,19^. As n-linked glycans show a strong heterogeneity among hosts, the feasibility of site-directed strategies based on enzymatic glycan labeling varies^18^. Site-specific labeling strategies based on protein tags or unnatural amino acids can only be applied to antibodies produced via recombination due to the necessary cloning step^20^. Therefore, non-site directed conjugation approaches are mostly used to build up larger libraries of oligonucleotide-conjugated antibodies. Several bioorthogonal conjugation methods have been established, where the most common are based on maleimide, tetrazine, or click chemistry reagents^14,21,22^. All these methods have in common the fact that the antibody and oligonucleotide have to be functionalized first with the respective reactive chemical group. Despite the vast variety of commercially-available crosslinking reagents and protocols for each of the non-site directed conjugation strategies, the oligonucleotide labeling of antibodies is not robust. The reason for this is multilayered, including problems related to a loss in the specificity of the antibody due to the masking of the antigen binding site^23^, a change in polarity upon the addition of the reactive conjugation group and oligonucleotide, and a lack of purification methods for the removal of excess oligonucleotides, which increases the rate of false positive errors. Another problem is that antibody-oligonucleotide conjugation is a consecutive reaction with a heterogeneous outcome of single-, multiple-, and non-labeled antibodies depending on the reaction conditions. The temperature, time, and molar stoichiometries of the antibody and oligonucleotide make the conjugation reaction a multiparameter optimization problem. The wide parameter space with low starting quantities of the antibodies makes it difficult to achieve acceptable conjugation efficiencies, and thus yields, in a research environment.

In this study, we established a protocol and analytical method by which to obtain single, double, and multiple oligonucleotide-conjugated antibodies. For the conjugation reaction between an antibody and an oligonucleotide, a state-of-the-art copper-free click chemistry reaction was performed between dibenzocyclooctyne (DBCO) and an azide. The antibodies were functionalized by crosslinking DBCO molecules to the amine side chains of the antibody via N-hydroxysuccinimide (NHS) functionalization. Since DBCO increases the hydrophobicity of the antibody, DBCO with four different crosslinkers was investigated to determine its conjugation efficiency and influence on antibody specificity. Oligonucleotides were functionalized with a counteracting azide group (3-azidopropionic acid sulfo NHS ester). Although the ring-strain promoted alkyne–azide [3 + 2] cyclo-addition between DBCO and azide is known to occur at physiological pH and room temperature (RT), we investigated the reaction kinetics under different temperatures and stoichiometric conditions in order to increase the product yield. We complemented our data by measuring the antibody conjugation efficiency in dependence of the oligonucleotide length and secondary structure. To purify and separate single- and double-oligonucleotide-conjugated antibodies, we developed an ion-exchange chromatography (IEX) protocol applicable with low entry sample volume. The advantages of using purified single, and double oligonucleotide-conjugated antibodies within immunofluorescence (IF) and proximity ligation assays (PLA) is demonstrated by their performance and specificity to unpurified and heterogeneously conjugated antibodies. Further, we demonstrate the need of single oligonucleotide-conjugated antibodies for methods using absolute read count statistics from next generation sequencing for protein quantification on the single cell level.

## Experimental Procedures

Chemicals were obtained from Sigma-Aldrich (Taufkirchen, Germany) unless stated otherwise. For the functionalization of the antibody we used dibenzocyclooctyne-N-hydroxysuccinimidyl ester (DBCO-NHS), dibenzocyclooctyne-sulfo-N-hydroxysuccinimidyl ester (DBCO-sulfo-NHS), dibenzocyclooctyne-S-S-N-hydroxysuccinimidyl ester (DBCO-S-S-NHS), dibenzocyclooctyne-PEG4-N-hydroxysuccinimidyl ester (DBCO-PEG_4_-NHS), and dibenzocyclooctyne-Cy5 (DBCO-Cy5). 3-Azidopropionic acid sulfo-NHS ester was purchased from Click Chemistry Tools (Scottsdale, AZ). Dialysis caps (Slide-A-Lyzer Mini) with a volume of 100 µl and 7 kDa MWCO molecular weight cut-off (MWCO), and desalting spin columns (Zeba), with a volume of 0.5 mL and 7 kDa MWCO, were obtained from Thermo Fisher Scientific (Munich, Germany). Ultracentrifugation spin columns (Amicon) with a volume of 0.5 mL and 3 and 10 kDa MWCO were obtained from Merck Millipore (Darmstadt, Germany).

### Antibody-crosslinker conjugation

The primary anti-Igfr-L1 (UniProt UPF0577) antibody was produced in rat (clone #16F6) and purified in the Monoclonal Antibody Core Facility of the Helmholtz Zentrum Munich. Anti-Igfr-L1 was an Immunoglobulin G (IgG). For conjugation, antibodies were adjusted to a concentration of 1 mg/ml with PBS. DBCO-NHS esters were dissolved in anhydrous DMSO, adjusted to 10 mM, and added to the antibody solution in varying molar excesses (from 1:1 to 1:25). The reaction was kept at RT for 45–60 minutes under rotary shaking. Unreacted and excessive DBCO-NHS was removed either by dialysis for 4 h at RT against PBS with a 10 kDa MWCO, gel-filtrated using desalting chromatography spin columns, or ultrafiltration using spin columns. The concentration of the antibody-DBCO conjugate was determined by bicinchoninic acid assay and QuBit Protein Assay both from Thermo Fisher Scientific (Munich, Germany). We measured and quantified the average number of conjugated DBCO molecules per antibody $({n}^{D-IgG})$ by absorption spectroscopy, using the following equation: ${n}^{D-IgG}=\frac{{c}^{D}}{{c}^{IgG}}$ (*c*^*D*^, where the DBCO (*c*^*D*^) and antibody (${c}^{IgG}$) concentration were obtained by ${c}^{D}=\frac{{A}_{309}}{{\varepsilon }_{309}^{D}}$ and ${c}^{IgG}=\frac{{A}_{280}^{c}}{{\varepsilon }_{280}^{IgG}}$, respectively. Here ${\varepsilon }_{309}^{D}$ and ${\varepsilon }_{280}^{IgG}$ are the molar extinction coefficients of the DBCO and IgG antibody at 309 nm (12,000 M^−1^ cm^−1^) and 280 nm (204,000 M^−1^ cm^−1^), respectively. ${A}_{309}$ is the absorption value of the sample at 309 nm. ${A}_{280}^{c}$ is the absorption value of the sample corrected by the absorption contribution of DBCO at 280 nm. ${A}_{280}^{c}\,$is calculated by ${A}_{280}^{c}={A}_{280}-({A}_{309}\cdot f)$ where the ${A}_{280}$ is the absorption value of the sample at 280 nm and *f* the correction factor of DBCO at 280 nm ($f=1.089$).

### Oligonucleotide-crosslinker conjugation

3-azidopropionic acid sulfo-NHS ester (3AA-NHS) was dissolved in anhydrous dimethyl sulfoxide (DMSO) at a concentration of 10 mM. Amine-modified oligonucleotides were dissolved in PBS at a concentration of 100 µM. 3AA-NHS in DMSO was added to the aqueous oligonucleotide solution to reach a 10 × molar excess of 3AA-NHS to oligonucleotide. The NHS reaction was kept at RT for 2 h under shaking conditions. Excess 3AA-NHS was removed either by dialysis for 4 h against PBS with a 7 kDa MWCO, by gel-filtration using a spin column with a 7 kDa MWCO, or by ultrafiltration by using spin filters with a 3 kDa MWCO. The concentration of the resulting azide-modified oligonucleotide was determined by absorbance spectroscopy at 260 nm using a microvolume UV/Vis spectrophotometer. For analytical purposes, we used an FPLC desalting column purchased from Thermo Fisher Scientific (89934). The column was run with PBS at a flow rate of 0.2 ml/min.

### Antibody-oligonucleotide conjugation

The azide-modified oligonucleotides were added to an antibody-DBCO solution (PBS, pH 7.2) under varying molar excesses, reaction temperatures, and incubation times in a microcentrifuge tube on a thermoshaker. The antibody-DBCO concentration in all reactions always ranged between 0.6–1 mg/ml.

### Purification of the antibody-oligonucleotide conjugate

Antibody-oligonucleotide conjugates were purified using ion exchange chromatography (Dynamic Biosensors, Martinsried, Germany). The analytical IEX column was purchased from Agilent (5190–2463; Waldkirch, Germany). The salt gradient for elution was started with 100% buffer A (50 mM Na_2_HPO_4_/NaH_2_PO_4_, 150 mM NaCl) and was gradually changed to 15.0% buffer A and 85.0% buffer B (50 mM Na_2_HPO_4_/NaH_2_PO_4_, 1 M NaCl) over the course of 16 min. While the analytical column was used at a flow rate of 0.5 ml/min, the preparative column was set to a flow rate of 1 ml/min. The collected fractions with antibody-oligonucleotide conjugates were concentrated using ultrafiltration columns with a 10 kDa MWCO. The yield of the antibody-oligonucleotide conjugates was determined by both a bicinchoninic acid assay and QuBit Protein assay.

### Cell culture

Min6 cells were cultured in DMEM with 10% FBS in T-25 flasks before transferring to fibronectin-coated glass-bottom plates (Corning 4581). Glass bottom plates were coated with 10 µg/ml fibronectin solution for 20 min, then washed with PBS. Cells were cultured on well plates for at least 24 h before the experiment or until a confluency of ~80% was reached.

### Immunofluorescence (IF)

Cells were fixed with 4% PFA in PBS (v/v) at RT for 15 min, then washed three times with PBS. Cell permeabilization was achieved with 0.5% Triton X-100 in 1% BSA/PBS for 30 min at 37 °C. The cells were then washed three times with PBS. Next, oligonucleotide-conjugated anti-Igfr-L1 was incubated for 1 h at RT. The samples were then washed 3 times with TBS-T before adding the secondary anti-rat IgG (A-21247; Thermo Fisher Scientific, Munich, Germany) in a 1:1000 dilution. After 1 h of incubation, the plate was washed three times with TBS-T, and subsequently stained with 1 ng/µl DAPI and 20 nM Phalloidin-Atto488 for 20 min.

### Proximity ligation assay

Cells were fixed and permeabilized as described previously. At all times, a liquid film of about 2–5 μL was allowed to cover the cells to prevent the sample from drying. The PLA assay was performed by adding the antibody-oligonucleotide conjugates for 1 h at RT at a concentration of 2–25 ng/μl. The cells were then washed 4 times with TBS-T for a total of 1 h. Ligation of the oligonucleotide strands was performed for 45 min at 30 °C in a 1 × T4 buffer solution containing 1 U/μl T4 DNA ligase, 1 × T4 buffer, 125 nM connector strands (see Table 1), 125 ng/ml BSA, and ddH_2_O. After washing three times with TBS-T, the rolling circle amplification was started by adding a 1 × phi29 buffer solution containing 0.25 U/μl phi29 polymerase, 200 μM dNTPs, and 200 μg/ml BSA in a microcentrifuge tube for 100 min at 32 °C with orbital shaking. Finally, the cells were washed twice with TBS-T and counterstained with a SSC solution containing 1 μg/mL DAPI, 6 nM detection probe for the PLA product, and 20 nM Phalloidin-Atto 488 for 30 min, before washing again three times with TBS.

**Table 1:** Oligonucleotides sequences.

Name	Sequence
O1	[AmC6dT]ACAACAACAAGAATGGAACCTCGCTAGAACGT
O2	[AmC6dT]ACAACAACAAGAATGGAACCTCGCTAGAACGT ACAACAACAAGAATGGAACCTCGCTAGAACGT
O3	[AmC6dT]ACAACAACAAGAATGGAACCTAGGTTCCATTC
O4	[AmC6dT] CAACAACAAAATAGTTCGGTCGAAGTTAGTCC
Connector 1	[Phos]AGGTTCCATTCAAAGGACTAACTTC
Connector 2	[Phos]GACCGAACTATCTAGTGCTGGATGATCGTCCC CCCTGCACCTCAAAACACCCTAACGTTCTAGCG
Probe	[Cy5]CTAGTGCTGGATGATCGTCC[Cy5]
O5	[AmC6dT] GTGACTGGAGTTCAGACGTGTGCTCTTCCGATCTG ACAGGAAGCTTTAAGGCCGGTCCTAGCAA

### Sequences of used oligonucleotides

All of the oligonucleotides used in the conjugations carried a 5′ amino modifier C6 dT, were double HPLC-purified, and were purchased from Ella Biotech. Desalted oligonucleotides used in PLA experiments were ordered from Sigma Aldrich (Taufkirchen, Germany).

### Image acquisition

Images for PLA dot counting were acquired with a Zeiss Observer Z1 epifluorescence microscope using a Plan Apochromat 20× (NA 0.8) objective and an Orca 12-bit camera (Hamamatsu) in μManager acquisition software^24^. In order to record the PLA dots throughout the entire Z-height of the cell, Z-stacks of 10 images with a step-size of 0.8 μm were acquired. The Z-stack was then transformed using a maximum projection. PLA dot detection was then performed by finding the local maxima within a grey level tolerance threshold. The threshold was set manually for images from one experimental series in dependence of the fluorescence background. The dot count was evaluated per nucleus. To achieve this, the DAPI-stained nuclei were segmented by applying a median filter and using a previously described iterative thresholding algorithm. A Voronoi diagram was then computed to segment the regions. The PLA dots per region were then counted. All image processing steps were carried out in ImageJ^25^. Images for publication were acquired using an 20× Plan Apochromat (NA 0.8) objective on a LSM800 microscope (Zeiss, Jena, Germany).

### CITE-seq

For the CITE-seq experiment anti-CD49e from R&D Systems (AF1864) was conjugated with oligonucleotide O5 using the DBCO-S-S-NHS ester linker. The oligonucleotide contains a reverse complement of Capture Sequence 1 of the 10x Genomics beads, an 8nt Feature Barcode and TruSeq Read 2. Within the CITE-seq run anti-CD49e was quantitative on human umbilical vein endothelial cells (HUVEC). HUVECs were maintained in endothelial growth medium (EGM) (AngioProteome cAP-02) on 1% gelatin-coated T25 flask to about 80% confluency before splitting. For the experiment HUVECS were detached from the culture plate using EDTA, washed in cold PBS twice, filtered through 40 µm cell strainers and their concentration adjusted to 1*10^6^ cells/ml. Cells were separated into two tubes, incubated with either the single- or the double oligonucleotide-conjugated anti-CD49e on ice for 30 min and again washed twice with PBS. The antibody concentration was 2 µg/10^6^ cells for each sample. Cells were then immediately processed for single cell droplet sequencing in the 10x Genomics Chromium device according to the manufacturer’s protocol (Single Cell Gene Expression with Cell Surface Protein, v3.1). 3ʹGene Expression Library Construction was omitted to only include Feature Barcodes. The cell surface protein library preparation was performed using the Single Index Kit T Set (10x Genomics, PN-1000213). The cell surface protein library was sequenced using a NextSeq 550 with a 10% spike-in of phiX to diversify the expected low heterogeneity of the library. Sequencing data was demultiplexed using cellranger. 10x Genomics barcodes with a Hamming distance of 1 were corrected. The barcode counts with unique UMIs were adjusted by the number of retrieved sequences per sample.

## Results and Discussion

The conjugation reaction of oligonucleotides to an antibody consists of three individual crosslinking steps: (i) functionalization of the antibody with a dibenzocyclooctyne (DBCO) click group; (ii) functionalization of the oligonucleotide with the corresponding azide click group; (iii) conjugation of the functionalized antibody and oligonucleotide via a copper-free click chemistry reaction. The functionalization of the antibodies and oligonucleotides with the click groups is achieved via NHS chemistry. In case of the antibody, the NHS ester is targeted using none-site-specific primary amines, for example lysine side chains. In case of the oligonucleotide, the NHS ester reacts with an amine modification at the 5′ end.

### Antibody and oligonucleotide functionalization

In order to maximize the yield of the antibody-oligonucleotide click chemistry conjugation reaction, we first sought to optimize the functionalization of the antibody and oligonucleotide. This was particularly important for the antibody functionalization for two reasons. Firstly, DBCO is hydrophobic, similar to the other click reactive groups, e.g. trans-cyclooctene. Thus, an increased number of DBCO molecules conjugated to the antibody reduces its solubility. A high number of DBCO moieties in the antibody may also change the specificity due to an increasing chance to label lysine residues at the binding domain of the antibody. Secondly, only a low fraction of DBCO groups on the antibody will react in the subsequent click reaction with an oligonucleotide due to the necessary alignment of the functional groups attached to two large polymers.

The low solubility of DBCO-NHS reportedly leads to the formation of turbid solutions during protein conjugation, which is an indication of the precipitation of reactants. Therefore, DBCO conjugation via NHS exhibits a low reproducibility and requires an analytical control of the conjugation products. For this reason, derivates of DBCO-NHS with variable soluble side groups and linkers have been developed. Linker length and their accompanied steric effects, however, are modulators of the copper-free click chemistry reactions. To investigate these factors in the NHS coupling reaction, but also in the click chemistry reaction, we included four different DBCO-NHS derivates in our test series.

**Figure 1:**
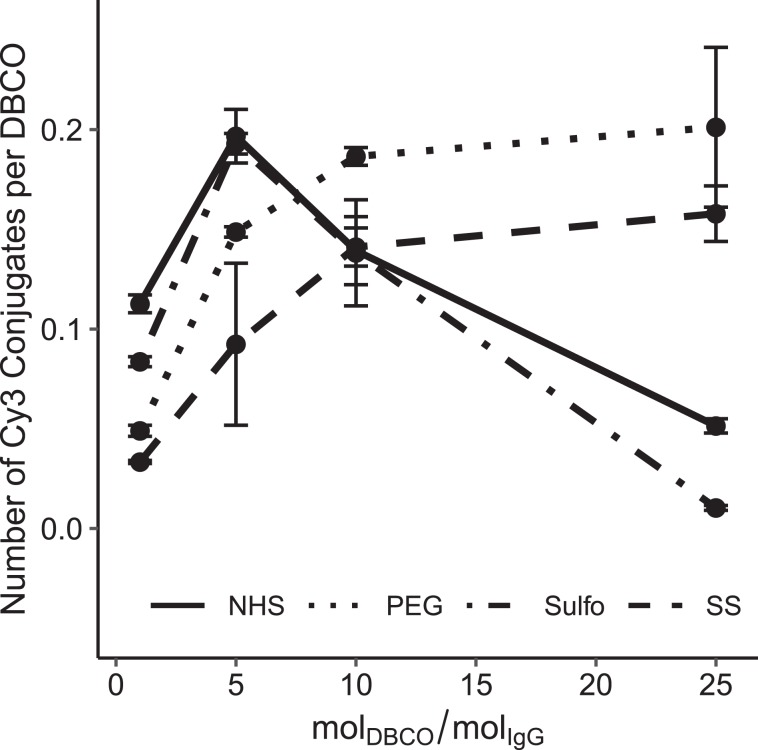
Click reaction yield in dependence of the molar ratio of DBCO-NHS to antibody used for the functionalization of the antibody. The average number of DBCO and Cy3 groups was determined by measuring the absorbance at 309 nm and 554 nm, respectively. After antibody functionalization, Cy3-azide was added at a 50 molar excess to saturate all functional DBCO groups. At a molar excess of about 5 to 10 mol DBCO per mol antibody, the reaction shows the highest conjugation yield in the subsequent click chemistry reaction for all tested DBCO derivates. Conjugation reactions with DBCO-Sulfo-NHS and DBCO-NHS with a molar ratio of DBCO to antibody above 5 resulted in protein and/or DBCO precipitation and, thus, a lower reaction yield. Error bars represent the standard error.

Figure 1 shows the conjugation yield of an azide fluorophore (N_3_-Cy3) to an IgG antibody functionalized with different numbers of DBCO-NHS, DBCO-Sulfo-NHS, DBCO-PEG_4_-NHS, and DBCO-SS-NHS molecules per antibody. The number of DBCO molecules on the antibody was adjusted by varying the molar ratio of DBCO-NHS and its derivates to the antibody in the functionalization step. The conjugate yield in the subsequent click reaction is expressed as the average number of Cy3-azide fluorophores coupled per the average number of DBCO groups on the antibody. This takes into account the fact that not all the DBCO groups on an antibody will react in the click reaction. At high DBCO/IgG ratios, the antibody-Cy3 conjugation yield is slightly higher for antibodies functionalized with DBCO-PEG_4_-NHS compared to DBCO-SS-NHS while the other two more hydrophobic derivates showed a low yield at higher molar ratios due to strong precipitation.

In contrast, the azide labeling of oligonucleotides with 3-azidopropionic acid (3AA-NHS) showed a maximum at a molar ratio of 3:1. All reactants are highly water soluble and thus no further optimization steps were needed.

Removal of excess click reagents after antibody and oligonucleotide functionalization was essential for increasing the yield of the subsequent click reaction by reducing the occurrence of unwanted reactions with left-over reactants. For this reason, we determined the removal and recovery yield of the three different purification methods with an entry volume of ≤100 µL, namely desalting, membrane ultrafiltration, and dialysis. The results are shown in Figs. S1 and S2 for the antibody and oligonucleotide NHS functionalization samples, respectively. Desalting and ultrafiltration were both performed with spin columns and removed a molar excess of 5 DBCO per antibody only to an extent of 82%, while retaining 90.1% and 82.3% of the antibody, respectively. After desalting a second time, only 12.2% of the DBCO remained in the solution, which resulted in an acceptable yield in the subsequent click conjugation reaction (see below); however, a large fraction of the antibody was lost. Dialysis lowered the DBCO to 13.2% of the initial content, while maintaining 94.6% of the antibody. Similar results were obtained for the purification of the oligonucleotide-azide functionalization reaction (Fig. S2). Therefore, hereafter, all antibody-DBCO and oligonucleotide-azide samples were purified by dialysis after click functionalization.

### Antibody-oligonucleotide click conjugation and purification

**Figure 2:**
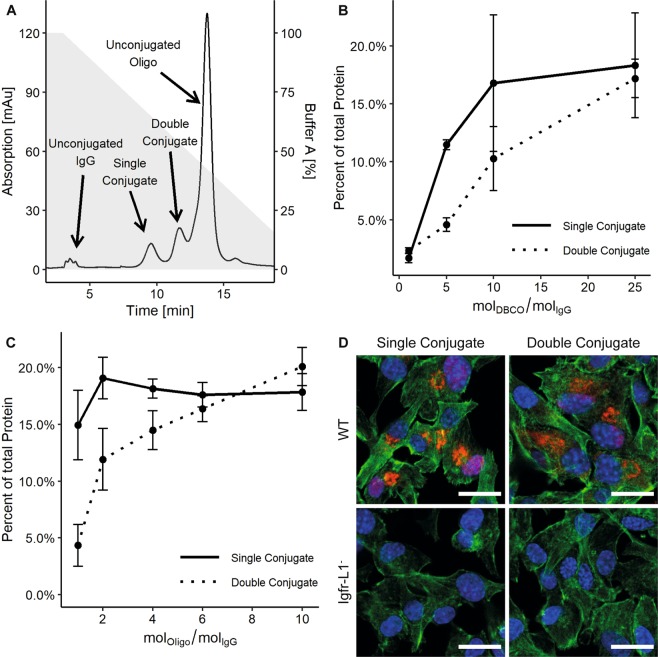
Ion exchange separation (IEX) of antibody-oligonucleotide conjugates. (**A**) Example of a typical IEX chromatogram of an antibody-oligonucleotide click conjugation reaction obtained with an anion exchange column at a flow rate of 1 ml/min. The gray area shows the salt gradient used for elution. For buffer conditions see Experimental Procedures. (**B**) Antibody-oligonucleotide click conjugation efficiency in dependence of the molar ratio of DBCO to antibody used in the functionalization reaction. (**C**) Antibody-oligonucleotide click conjugation efficiency in dependence of molar oligonucleotide excess at constant molar ratio of DBCO to antibody. Error bars represent standard error. (**D**) IF images of the oligonucleotide-conjugated anti-Igfr-L1 antibody in wild type and anti-Igfr-L1 knock out Min6 cells. Scale bar: 20 µm.

The click reaction between functionalized antibodies and oligonucleotides is a consecutive second-order reaction. The yield and product types of the click reaction are dependent on: (i) number of DBCO molecules on the antibody; (ii) the molar ratio of the functionalized antibody to oligonucleotide; (iii) the conjugation time; (iv) the reaction temperature. For example, increasing the number of DBCO molecules on the antibody may lead to antibodies being conjugated to two or more oligonucleotides instead of one. Therefore, we aimed to find the optimal click reaction conditions to maximize the yield of antibodies with a single conjugated oligonucleotide. In order to differentiate but also purify unconjugated, single, and multiple conjugated antibody-oligonucleotides, the click reaction samples were separated using ion-exchange chromatography (IEX). Figure 2A shows a representative IEX chromatogram recorded at an absorption wavelength of 260 nm. Unconjugated antibodies have the lowest negative net charge and, thus, eluted first from the anion exchange column, followed by the single and double oligonucleotide-conjugated antibodies. The unconjugated oligonucleotides were last to be eluted from the column. The number of oligonucleotide conjugates per antibody in the different fractions was confirmed by SDS gel chromatography (see Fig. S4).

Using this separation technique, we started to optimize the click reaction by determining the influence of the number of DBCO molecules in the antibody on the conjugation yield. The number of DBCO molecules per antibody was adjusted by varying the molar ratio of DBCO to antibody during functionalization. The molar ratio of oligonucleotide to antibody was kept constant at 3. The conjugation yield for single and double oligonucleotide-conjugated antibodies was calculated by determining the protein concentration of the individual IEX peaks. The results are provided in Fig. 2B. While the yield of single oligonucleotide-conjugated antibodies reached saturation at a molar ratio of 10 (DBCOs/IgG), the amount of double conjugates continued to increase for higher ratios. The offset of the molar ratio for double compared to the single oligonucleotide-conjugated antibodies was expected due to its dependence on the presence of single conjugates.

Next, the molar ratio of oligonucleotide to antibody was optimized to increase the yield of single oligonucleotide-conjugated antibodies. For this, only the antibodies functionalized with a molar DBCO/antibody ratio of 10 were used. Figure 2C shows these results. The saturation of single oligonucleotide-conjugated antibodies was already obtained at a molar ratio of two. Notably, even for high molar ratios of oligonucleotide to antibody, the double oligonucleotide-conjugated antibodies were not saturated. For the next optimization steps, the molar ratio of oligonucleotide to antibody in the click reaction was kept constant at 3.

An essential question within the antibody DBCO functionalization and oligonucleotide conjugation reactions is whether the antibody maintains its specificity. To evaluate the specificity of the antibodies with PLA, single and double conjugates of O1 and anti-Igfr-L1 were created with molar ratios of 10 DBCO/IgG and 3 oligonucleotides/IgG. Figure 2D shows the corresponding PLA images in Min6 wild type cells and Min6 cells with knocked out Igfr-L1 as control. The Igfr-L1 receptor has been previously shown to localize to the endoplasmic reticulum (ER). All antibodies maintained their specificity and dots were highly localized close to the nucleus in the ER region, while the knock out cells showed no signal. Upon changing the crosslinker chemistry, the specificity of the antibody did not change, although the best quality of the IF images was obtained with oligonucleotide-conjugated IgG carried an DBCO-PEG_4_ linker (see Fig. S3).

**Figure 3:**
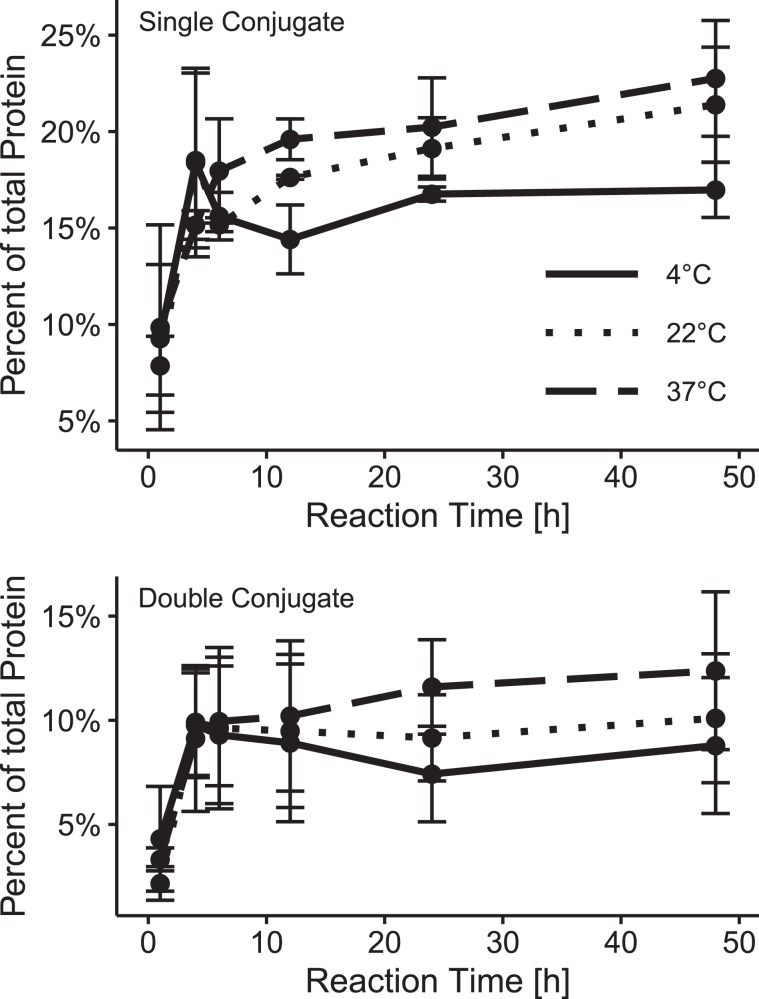
Temperature dependence of the oligonucleotide/antibody click conjugation kinetic. (**A**,**B**) Click reaction kinetics for the single and double oligonucleotide conjugation products over the course of 48 h for 4, 22, and 37 °C, respectively. Antibody yields were calculated from the chromatograms of IEX separations.

Next, the optimal reaction time and temperature for the click reaction between the antibody and oligonucleotide was determined. For this, the molar ratio of DBCO to antibody in the functionalization reaction was set to 10 and the molar ratio of the oligonucleotide to antibody to 3 within the click reaction. The click reaction kinetics for single and double oligonucleotide-conjugated antibodies at 4, 22, and 37 °C are provided in Fig. 3. A steady state level of the consecutive conjugation reaction was observed for all the reaction temperatures after 10 and 4 h for the single and double oligonucleotide-conjugated antibody, respectively. As the temperature increased, the yield of the antibodies also increased. This indicates that 37 °C is a physiological temperature, however, the long reaction time may influence the stability of the antibody and thus its specificity. Whether high temperatures and long incubation times reduce the specificity of the antibody was tested by IF. We did not observe any changes in specificity.

### Influence of oligonucleotide length and secondary structure on the click conjugation yield

**Figure 4:**
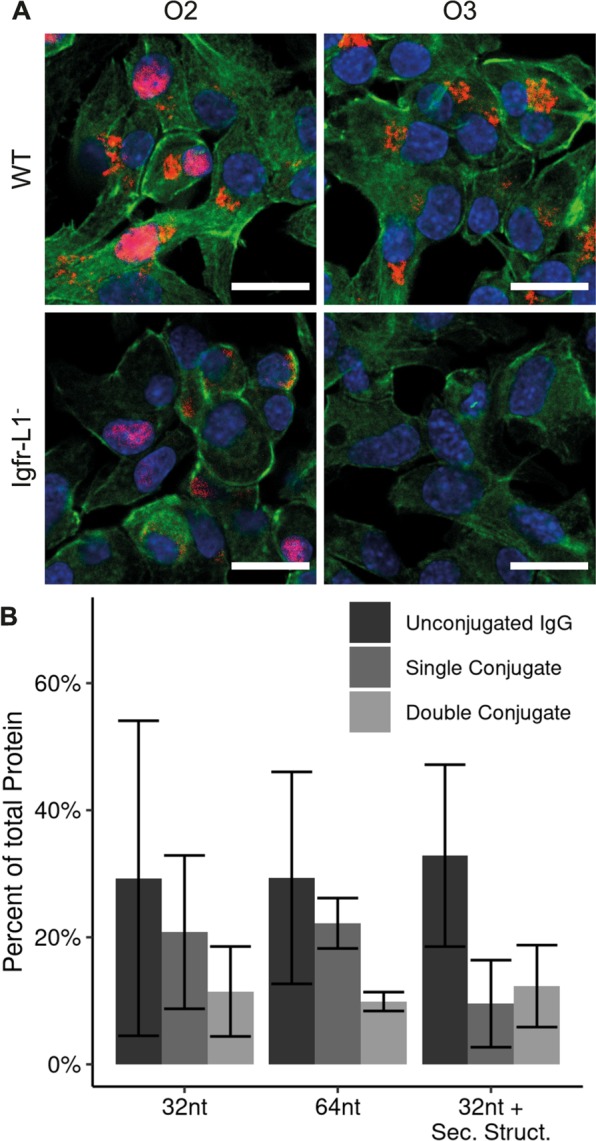
Influence of the oligonucleotide length and secondary structure on the antibody-oligonucleotide conjugation yield and specificity. (**A**) IF images of single O2 and O3 conjugated anti-Igfr-L1 in wild type and Igfr-L1 knock out Min6 cells. Scale bar: 20 µm. (**B**) Comparison of the influence of oligonucleotides with different compositions on the conjugation yield. Comparing oligonucleotides with a length of 32 nt and 64 nt, no conjugation differences were observed. A secondary structure within the oligonucleotide reduced the fraction of single oligonucleotide conjugate antibody by about 56%. Error bars represent standard error.

Depending on the application of antibody-oligonucleotide conjugates, the length and sequence of the oligonucleotide can change. Therefore, we investigated whether the oligonucleotide length or structure elements within the oligonucleotide influence the conjugation efficiency. For this, the previously determined parameters for NHS functionalization and the click conjugation reactions were kept constant. We used two oligonucleotides with 32 (O1) and 64 nucleotides (nt), where the longer oligonucleotide contained two sequence repeats of the 32 nt oligonucleotides (O2). The sequence of the 32 nt oligonucleotide was designed by minimizing its free energy for the secondary structure. The repeat of the 32 nt sequences showed equally low theoretical free energy values for the secondary structure formation. Figure 4B shows the click reaction efficiencies for obtaining single and double oligonucleotide-conjugated antibodies. In fact, we observed no differences in this length regime. However, when we included a hairpin structure of 11 nt at the 3′ end of a 32 nt long oligonucleotide (O3), the coupling efficiency for single oligonucleotide-conjugated antibodies decreased to 44.1%. Notably, the hairpin was designed to not mask the free azide group of the oligonucleotide at the 5′ end. Single and double oligonucleotide-conjugated antibodies with the 32 nt, 64 nt, and 32 nt oligonculeotide with hairpin structure localized correctly at the ER, as tested by IF staining, in Min6 cells (see Fig. 4A). However, we observed a higher background fluorescence along with low fluorescence in the knock out cells for the 64 nt oligonucleotide conjugate.

In order to demonstrate that our oligonucleotide conjugation approach is of general use and not specific to anti-Igfr-L1, we labeled 19 different antibodies under the optimized click reaction conditions with 18 different oligonucleotide, respectively. The oligonucleotide length ranged between 32 nt and 43 nt. The average conjugation yield for the single and double oligonucleotide-conjugated antibodies was 18 and 8%, respectively, of the entry antibody mass (see Fig. S5).

### Proximity ligation assay with single and double oligonucleotide-conjugated antibodies

To demonstrate the functionality and advantage of using purified antibody-oligonucleotide conjugates, we performed a PLA. In a PLA, antibodies bind to their targets within a fixed cell. Their proximity is tested by the addition of connectors bridging the two oligonucleotide conjugates. Two connectors are used to form a circular DNA template that can be amplified by rolling circle amplification. The product is an ssDNA polymer with a diameter of ∼0.5 μm that is detectable by fluorescence microscopy at low magnification. We previously noted a large variation in the PLA results between batches of self-conjugated antibodies. We hypothesized that one reason for this may be the unoptimized conjugation parameters. Another reason could be the purity of the antibody-oligonucleotide solution, since, in previously described protocols for antibody oligonucleotide conjugation reactions, the excess of unconjugated oligonucleotides was not efficiently removed, where the remaining oligonucleotides can lead to false-positive signals.

**Figure 5:**
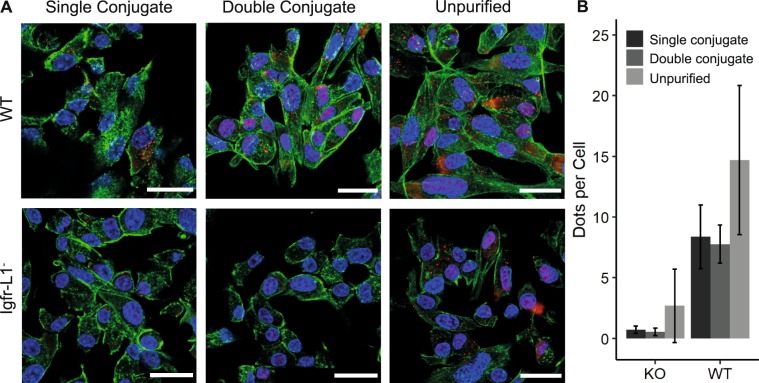
Proximity ligation assay (PLA) with purified single and double oligonucleotide-conjugated antibodies compared to unpurified oligonucleotide-conjugated antibodies. (**A**) Fluorescence images of wild type Min6 cells with PLA dots (red) generated by targeting the homodimer receptor Igfr-L1. Nucleus and actin cytoskeleton of the Min6 cells were counterstained with DAPI (blue) and phalloidin (green). Scale bar: 20 µm. (**B**) PLA dot counts per cell to the corresponding fluorescence images in A. Samples size: 8180 cells. Error bars represent standard deviation.

The Igfr-L1 receptor forms a homodimer. To generate PLA signals with single and double oligonucleotide-conjugated anti-Igfr-L1, the antibody was conjugated once with oligonucleotides O1 and O4. Figure 5B shows the PLA dot count per cell for the single and double oligonucleotide-conjugated anti-Igfr-L1 in wild type Min6 cells. Both anti-Igfr-L1 conjugates show comparable PLA dot counts per cell. In Min6 cells with the Igfr-L1 KO, the purified anti-Igfr-L1 conjugates showed no signal. The unpurified anti-Igfr-L1 conjugates exhibited a significantly higher PLA dot count in WT and KO Min6 cells, a reduced ER localization, and a higher cell-to-cell variability (see Fig. 5A).

### Single and double conjugated-oligonucleotide antibodies in quantitative sequencing read count statistics

**Figure 6:**
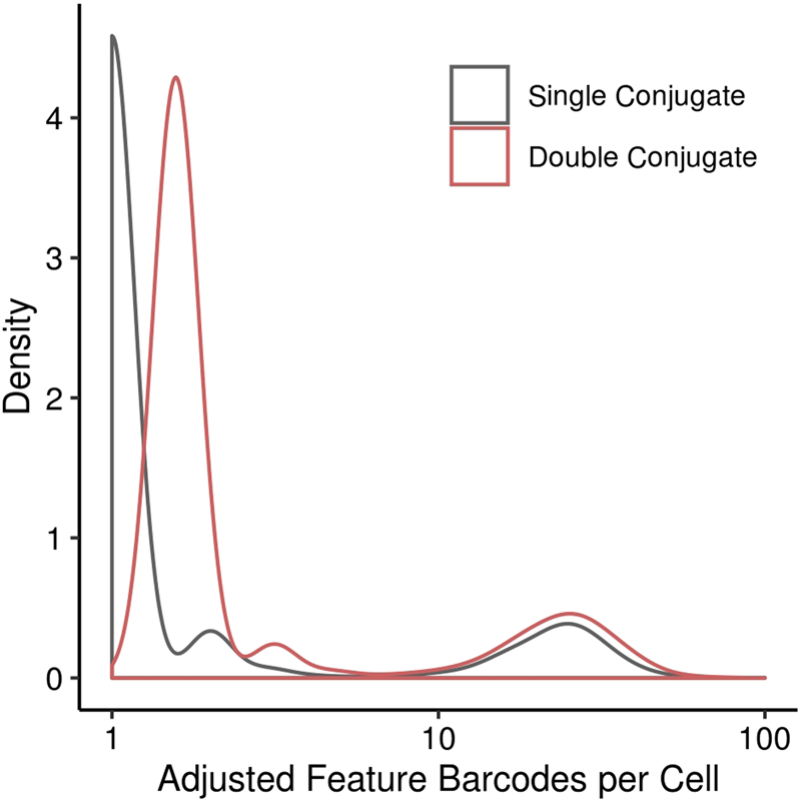
Distribution of feature barcode binding events per single cell in a CiteSeq experiment with single and double oligonucleotide-conjugated anti-CD49e. Double oligonucleotide-conjugated anti-CD49e increase the number of retrieved sequencing reads per HUVEC. The number of reads per cell was corrected for UMI duplication and adjusted by the total read number per sample.

To demonstrate the influence of the oligonucleotide number on an antibody within quantitative measurements of proteins with help of read count statistics from next generation sequencing approaches, we performed a CITE-seq experiment. For this, single and double oligonucleotide-conjugated anti-CD49e were generated following the optimized conjugation protocol given for anti-Igfr-L1 in Fig. 5, with the difference that a DBCO-SS-NHS ester was used instead of an DBCO-NHS ester linker. Anti-CD49e was detected on the surface of single HUVECs by using 10x Genomics droplet technology. For this, two HUVEC samples, one stained with single and another one with double oligonucleotide-conjugated anti-CD49e were encapsulated into a water/oil droplet of the 10x Genomics Chromium platform. Within the reducing environment of the droplets the conjugated oligonucleotides are released from the antibody, captured, and barcoded to assign the oligonucleotide to one particular cell. A unique molecular identifier (UMI) sequence, along with a cell specific barcode added to the conjugated oligonucleotide sequence allows to count single antibody binding events on a cell by exploiting read count statistics from next generation sequencing technology. Figure 6 shows the density plot of adjusted feature barcode reads per cell for the single and double conjugated anti-CD49e from single HUVCES cells. Following the expectation we find that double oligonucleotide-conjugated anti-CD49e leads to a doubling of the sequencing read count statistics. The low read number per cell is explained by the low sequencing depth of 4.1 and 2.6 million mapped reads for the single and double oligonucleotide-conjugated anti-CD40e sample, respectively.

## Conclusion

In this study, we developed and optimized a workflow to obtain purified antibodies with defined numbers of conjugated oligonucleotides. Upon establishing the IEX purification of the reaction products, we systematically optimized the parameters of the copper free click chemistry conjugation reaction between an IgG antibody and oligonucleotides. The presented workflow allows to conjugate antibody quantities down to 25 ng with a yield of 5 ng (20%) single oligonucleotide-conjugated IgG antibody. Thus, the method presented here is cost-effective and suitable for the conjugation of antibody quantities available from commercial sources. However, it is worth noting that this only holds true when no amine-containing additive is used in the formulation of the antibody.

In contrast to previous oligonucleotide to antibody conjugation protocols, we validated all antibody conjugates against knock out controls for their specificity and prove general applicability^17,26^. This allowed for the optimization of all parameters with regards to the antibody specificity. In particular, we observed that our test antibody conjugated to oligonucleotides with a length of 64 nt exhibited an increased background signal in IF and PLA compared to the same antibodies conjugated with an oligonucleotide of 32 nt length. This finding holds true for 19 other antibodies but still may slightly vary for others. Importantly, for the design of oligonucleotides for antibody conjugation, we showed that structured oligonucleotides exhibit a lower conjugation yield compared to unstructured oligonucleotides with the same length. Therefore, techniques requiring hairpins or toeholds for signal detection must consider a higher starting quantity of antibodies. The different crosslinkers for functionalized antibodies with DBCO had no influence on the specificity within the IF and PLA experiments. However, to obtain comparable reaction yields with more hydrophobic crosslinkers for DBCO, the molar ratio between DBCO and antibody has to be kept ≤10, which is far lower than previously used molar ratios for protein functionalization.

In conclusion, the importance of the purification of oligonucleotide-conjugated antibodies is apparent for proximity ligation assays. After removing the excess oligonucleotides, the PLA signal for the homodimer formation of anti-Igfr-L1 negative controls in a knock out cell line showed no signal, whereas the unpurified antibodies showed a background signal. PLA has been reported to be error-prone due to their long processing steps. By including a step for the purification of the reaction components, we believe that we can increase the reliability of the detection system for protein interactions. For quantitative protein detection via amplification methods and/or absolute read count statistics by next generation sequencing not only the purity of oligonucleotide-conjugated antibodies is of importance but also the control of the precise number of conjugated oligonucleotides. This we demonstrated by using single and double oligonucleotide-conjugated anti-CD49e antibodies in a CITE-seq experiment. According to our expectations the read numbers for antibodies increased with double-conjugated oligonucleotides by a factor of two. Importantly, all current CITE-seq protocols do not include a control of the number of conjugated-oligonucleotides per antibody^12,27^. None-site-directed antibody conjugation strategies lead to mixed antibody products with varying numbers of conjugated oligonucleotides. In consequence CITE-seq results are biased in their read count statistics and in particular contain a large variation between experiments when not using the same batch of conjugated antibodies.

In summary, the method presented here could be used to increase the popularity of the use of oligonucleotide-conjugated antibodies. With these improvements, it would be of interest to combine the use of single oligonucleotide-conjugated antibodies with multiplexed CITE-seq screens, to validate the increased robustness of the protein quantification method.

## Supplementary information

Supplementary informations.
