# Supplementary material for: Preparation of single- and double-oligonucleotide antibody conjugates and their application for protein analytics
Source: Sci Rep. 2020 Jan 29;10:1457. doi: 10.1038/s41598-020-58238-6 (PMC6989672; doi:10.1038/s41598-020-58238-6)
Supplement: Supplementary file 1 — Supplementary informations. [file 41598_2020_58238_MOESM1_ESM.docx]

**Supplementary Information**

**Preparation of single- and double-oligonucleotide antibody conjugates and their application for protein analytics**

Julius Wiener^†^, Daniel Kokotek^†^, Simon Rosowski^†^, Heiko Lickert^‡^, Matthias Meier^†*^

^†^Microfluidic and Biological Engineering, Helmholtz Pioneer Campus, Helmholtz Zentrum München, Ingolstaedter Landstr. 1, 85764 Neuherberg, Germany

^‡^Institute of Diabetes and Regeneration Research, Helmholtz Zentrum München, D-85764 Neuherberg, Germany

^‡^German Center for Diabetes Research (DZD), D-85764 Neuherberg, Germany

^‡^Institute of Stem Cell Research, Helmholtz Zentrum München, D-85764 Neuherberg, Germany

^‡^Technical University of Munich, School of Medicine, Munich, Germany

^*^Correspondence should be addressed to: matthias.meier@helmholtz-muenchen.de

Figure S1. Comparison of purification methods by antibody recovery and removal of excess DBCO-NHS after functionalization. Anti-Igfr-L1 (1mg/ml) was conjugated with DBCO-Sulfo-NHS at a molar ratio of 1:5 (IgG/DBCO) for 1 h in PBS at RT. Antibody recovery efficiencies were determined by measuring the protein concentrations before and after purification with BCA. The amount of remaining DBCO-Sulfo-NHS was determined with a desalting FPLC column, where the DBCO-Sulfo-NHS could be separated. The integrals of the absorption band at 260 nm of free DBCO in unpurified and purified samples were used to calculate the purification efficiency. While the lowest amount of free DBCO-Sulfo-NHS (12.2%) was obtained upon performing two sequential desalting spin column runs, the highest recovery rate, while still achieving high clean-up efficiencies, was obtained by dialysis at a 7 kDa MWCO.

Figure S2. Comparison of oligonucleotide recovery and purification methods for the removal of excess click functionalization groups. The oligonucleotide (O1) was conjugated with a molar ratio of 1:50 (oligonucleotide/3AA-NHS) for 1 h at RT. The oligonucleotide recovery efficiency was determined by absorbance spectrometry at 260 nm. The amount of remaining 3AA-NHS was determined in an FPLC experiment. The peak corresponding to 3AA-NHS was integrated and compared to the unpurified sample. While the lowest amount of free 3AA-NHS (4.53%) was obtained by desalting the reaction product two times with spin columns (7kDa MWCO), the highest recovery rate was achieved with an ultrafiltration spin column (10 kDa MWCO). The purification efficiency for the ultrafiltration spin columns, however, was low. Dialysis and desalting spin columns were equally effective in terms of oligonucleotide recovery and purification efficiency.

Figure S3. IF images of single oligonucleotide-conjugated Igfr-L1 using different DBCO crosslinkers. While the expected localization of the Igfr-L1 at the ER was observed for all conjugated antibodies in wild type Min6 cells, the control experiments using Igfr-L1 knock out Min6 cells were negative.

Figure S4. SDS gel of the single and double oligonucleotide-conjugated Igfr-L1. (A, B) Gel stained with SYBR Gold and silver staining, respectively.


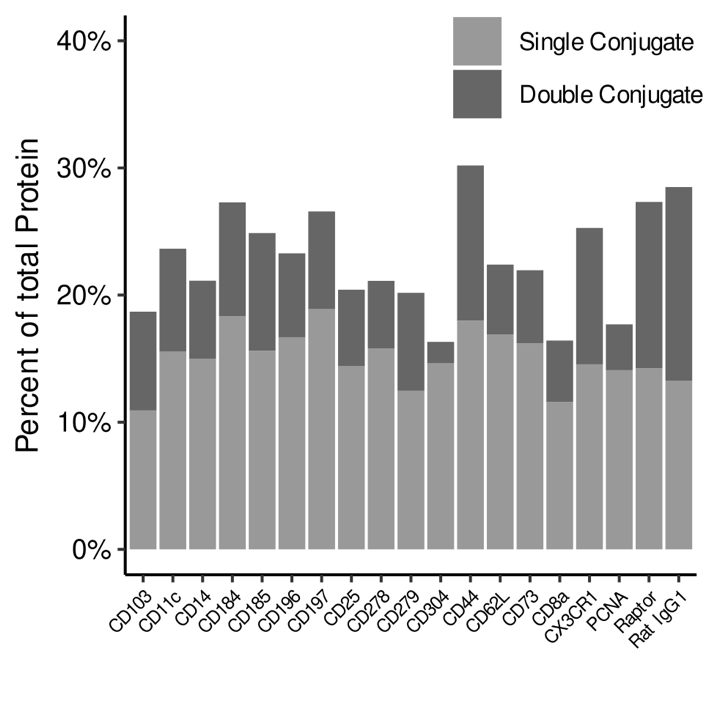


Figure S5. Oligonucleotide-antibody conjugation efficiency for 19 different antibodies and oligonucleotides, respectively. The corresponding oligonucleotide sequences are given in table S1. All conjugation reaction were performed with an 5:1 DBCO-S-S-NHS to Ab molar ratio within a click reaction of 4h at room temperature. The conjugation protocol for all Ab resembles the protocol for the single and double oligonucleotide conjugated anti-Igfr-L1 in figure 5.

Table S1: Oligonucleotide sequences corresponding to Figure S5.

| Antibody Name | Conjugated Sequence |
| --- | --- |
| CD 103 | CGGAGATGTGTATAAGAGACAGGCAGAGAACCCATATAAGAAA |
| CX3R1 | CGGAGATGTGTATAAGAGACAGGAGTAGACCCCATATAAGAAA |
| CD 62L | CGGAGATGTGTATAAGAGACAGAGTGATGCCCCATATAAGAAA |
| CD 14 | CGGAGATGTGTATAAGAGACAGAAGAACCGCCCATATAAGAAA |
| CD 196 | CGGAGATGTGTATAAGAGACAGAGACGGAACCCATATAAGAAA |
| CD 73 | CGGAGATGTGTATAAGAGACAGAGCGAACACCCATATAAGAAA |
| CD 278 | CGGAGATGTGTATAAGAGACAGTTAGTGGCCCCATATAAGAAA |
| CD 19 | CGGAGATGTGTATAAGAGACAGGAACTGGACCCATATAAGAAA |
| CD 184 | CGGAGATGTGTATAAGAGACAGAACCAAGGCCCATATAAGAAA |
| CD 185 | CGGAGATGTGTATAAGAGACAGCGATTGACCCCATATAAGAAA |
| CD 197 | CGGAGATGTGTATAAGAGACAGAACCTGACCCCATATAAGAAA |
| CD 44 | CGGAGATGTGTATAAGAGACAGTGTTGACCCCCATATAAGAAA |
| CD 4 | CGGAGATGTGTATAAGAGACAGGACAGGAACCCATATAAGAAA |
| CD 25 | CGGAGATGTGTATAAGAGACAGAAGGCGAACCCATATAAGAAA |
| CD 8a | CGGAGATGTGTATAAGAGACAGCGACAAGACCCATATAAGAAA |
| CD 279 | CGGAGATGTGTATAAGAGACAGACGATGAGCCCATATAAGAAA |
| CD 11b | CGGAGATGTGTATAAGAGACAGCGAGCTAACCCATATAAGAAA |
| CD 183 | CGGAGATGTGTATAAGAGACAGACCAAGGACCCATATAAGAAA |
| CD 304 | CGGAGATGTGTATAAGAGACAGTGCGTGAACCCATATAAGAAA |
| CD 11c | CGGAGATGTGTATAAGAGACAGAGACTACGCCCATATAAGAAA |
